# Effect of Cold-Rolling Deformation on the Microstructural and Mechanical Properties of a Biocompatible Ti-Nb-Zr-Ta-Sn-Fe Alloy

**DOI:** 10.3390/ma17102312

**Published:** 2024-05-14

**Authors:** Vasile Dănuț Cojocaru, Alexandru Dan, Nicolae Șerban, Elisabeta Mirela Cojocaru, Nicoleta Zărnescu-Ivan, Bogdan Mihai Gălbinașu

**Affiliations:** 1 Faculty of Materials Science and Engineering, National University of Science and Technology Politehnica of Bucharest, 060042 Bucharest, Romania; dan.cojocaru@upb.ro (V.D.C.); alexandru.dan0806@upb.ro (A.D.); nicolae.serban@upb.ro (N.Ș.); elisabeta.cojocaru@upb.ro (E.M.C.); 2 Dental Medicine Faculty, University of Medicine and Pharmacy “Carol Davila” Bucharest, 020021 Bucharest, Romania; bogdan.galbinasu@umfcd.ro

**Keywords:** mechanical properties, microstructure, β-Ti phase, cold deformation, SEM analysis, XRD analysis, titanium alloy

## Abstract

The primary focus of the current paper centers on the microstructures and mechanical properties exhibited by a Ti-30Nb-12Zr-5Ta-2Sn-1.25Fe (wt. %) (TNZTSF) alloy that has been produced through an intricate synthesis process comprising cold-crucible induction in levitation, carried out in an atmosphere controlled by argon, and cold-rolling deformation (CR), applying systematic adjustments in the total deformation degree (total applied thickness reduction), spanning from 10% to 60%. The microstructural characteristics of the processed specimens were investigated by SEM and XRD techniques, and the mechanical properties by tensile and microhardness testing. The collected data indicate that the TNZTSF alloy’s microstructure, in the as-received condition, consists of a β-Ti phase, which shows polyhedral equiaxed grains with an average grain size close to 82.5 µm. During the cold-deformation processing, the microstructure accommodates the increased applied deformation degree by increasing crystal defects such as sub-grain boundaries, dislocation cells, dislocation lines, and other crystal defects, powerfully affecting the morphological characteristics. The as-received TNZTSF alloy showed both high strength (i.e., ultimate tensile strength close to σ_UTS_ = 705.6 MPa) and high ductility (i.e., elongation to fracture close to ε_f_ = 11.1%) properties, and the computed β-Ti phase had the lattice parameter a = 3.304(7) Å and the average lattice microstrain ε = 0.101(3)%, which are drastically influenced by the applied cold deformation, increasing the strength properties and decreasing the ductility properties due to the increased crystal defects density. Applying a deformation degree close to 60% leads to an ultimate tensile strength close to σ_UTS_ = 1192.1 MPa, an elongation to fracture close to ε_f_ = 7.9%, and an elastic modulus close to 54.9 GPa, while the computed β-Ti phase lattice parameter becomes a = 3.302(1) Å.

## 1. Introduction

Ever since it was first used on a large industrial scale in the 1950s, Titanium has seen widespread use in an extensive array of domains, from the automotive and airspace to chemical, nuclear and biomedical applications. Titanium, by itself, has an impressive set of properties, from a high melting point of 1725 °C and ease of casting and forging, to a high corrosion resistance, to having a low density when compared to iron. While providing a higher tensile strength, it is often enough ill-suited, in and of itself, for many applications. As such, it is usually allied with other elements, in order to obtain a new material with better properties, one that is more adequate for the task at hand [1,2,3,4].

One area of particular interest for titanium alloys has been the biomedical field, specifically within the domain of orthopaedic implants [5,6,7]. In order to be considered for implant applications, a material must possess a high biocompatibility, so as to avoid being rejected by the body, and to not have toxic or carcinogenic properties. Moreover, implant materials are expected to have good mechanical compatibility with the human body: they must resist friction and wear, as well as multiaxial fatigue loading, and have a high tensile strength, good ductility, and a Young’s modulus that is close to that of the human bone, so as to avoid the stress shielding effect. Furthermore, the human body presents a very corrosive medium, which the orthopaedic implant must handle. Titanium alloys, especially those containing the β-Ti phase, are uniquely suited to meeting these requirements [8,9,10,11,12]. Nb, Ta, and Zr are considered the safest, most non-toxic alloying elements for biomedical applications, and also provide a final material that has the required mechanical and corrosion resistance properties and good tissue compatibility [13].

A further way to improve this already good class of materials is through either thermomechanical or mechanical processing, as it was shown that there is a direct correlation between the alloy’s microstructure and its mechanical properties, with grain size having a particularly large impact on said characteristics. Many studies have shown that these two processing processes (mechanical and thermomechanical) have the potential to improve the mechanical properties of Ti-based alloys, thus making them suitable to be used as implantable materials [1,14,15].

As for Ti-based alloys, deformation by cold-rolling is the most widely used mechanical process, which plays an important role in tailoring its mechanical characteristics. Through deformation by cold-rolling, significant changes can be achieved in the alloy’s microstructure, such as in its grain refinements, thus lessening residual stress or increasing dislocation density; all of this leads to improving its mechanical strength and hardness.

Although over time a lot of work has focused on finding the right mechanical processing route to acquire the best mechanical properties for Ti-based alloy, none of them have found a perfect balance; as such, designing a fitting mechanical processing route is very important for obtaining a final material with better properties than that of as-cast alloy.

Consequently, this work examines the effects of deformation by cold-rolling on a Ti-30Nb-12Zr-5Ta-2Sn-1.25Fe (wt. %) (TNZTSF) alloy. The as-received TNZTSF alloy was subjected to varying deformation degrees, ranging from ε = 10% to ε = 60% in 10% increments, and then analyzed microstructurally and mechanically, so as to determine which deformation degree yields the best results.

## 2. Materials and Methods

### 2.1. The Alloy’s Synthesis

Our studied alloy Ti-30Nb-12Zr-5Ta-2Sn-1.25Fe (wt. %), referred to as TNZTSF, was synthesized using a cold crucible FIVE CELES-MP25 levitation induction furnace, in a controlled argon atmosphere, in order to avoid the formation of oxides. To ensure a high level of chemical homogeneity, the alloy underwent three cycles of remelting. The alloy was synthesized using just high-purity elemental components such as titanium (min. 99.6%), niobium (min. 99.9%), zirconium (min. 99.5%), tantalum (min. 99.9%), tin (min. 99.96%), and iron (min. 99.98%).

### 2.2. Mechanical Processing Route

**Figure 1:**
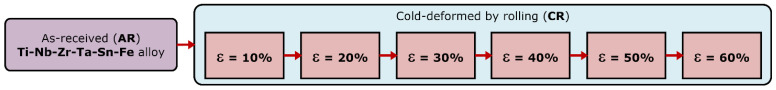
Processing scheme applied to Ti-30Nb-12Zr-5Ta-2Sn-1.25Fe (TNZTSF) (wt. %) alloy.

Displayed in Figure 1 is the utilized mechanical processing route, beginning with the as-received TNZTSF alloy, which was then deformed by cold rolling with various deformation degrees, ranging from ε ≈ 10% to ε ≈ 60%, in 10% increments. The cold rolling steps were carried out with the aid of a MARIO di MAIO LQR120AS rolling mill.

These mechanical processing steps were carried out with the objective of inducing modifications in the TNZTSF alloy’s microstructure and assessing the impacts of different deformation degrees on it, in order to gauge the best possible mechanical processing route for obtaining an optimal microstructure, with a small grain size, and a good fusion of mechanical properties (high strength and ductility, combined with a low elastic modulus) that would make a final material ideally suited for biomedical implant applications.

### 2.3. Microstructural and Mechanical Analysis of the Alloy

The as-received (AR) alloy, together with the alloys that were cold-deformed by rolling (CR), were subjected to both microstructural and mechanical analyses to gauge the impact of the chosen mechanical processing routes on the TNZTSF alloy’s microstructure. Specimens were sectioned from each state with the aid of a precision METKON Micracut 202 cutting machine. This machine was equipped with an NX-MET XDLM ∅150 mm diamond cutting disk. After the cutting procedure, the samples were hot-mounted using an NX-MET XPHC carbon conductive phenolic resin, facilitated by the advanced BUEHLER SimpliMet2 hot-mounting press.

Both sample preparation techniques (grinding and polishing) were carried out using a METKON Digiprep ACCURA machine. For the first sample preparation technique, namely, grinding, we utilized the NX-MET XPAC ∅250 mm SiC abrasive papers in different 5-stage routines, ranging from 180 to 1200 grit, while for the second sample preparation technique, polishing, we utilized NX-MET M200 ∅250 mm soft flocked polishing cloths, as well as 6 and 1 μm consecutive applications of NX-MET XP15 polycrystalline diamond suspensions. The final polishing process entailed the use of a NX-MET M100 ∅250 mm buffet polyurethane cloth and NX-MET XA05 0.05 μm colloidal silica fused with 20% H_2_O_2_. In order to be able to perform SEM-EBSD investigations, an extra super-polishing procedure was implemented using a BUEHLER VibroMet2 vibro-polishing machine, utilizing the NX-MET M210 ∅300 mm soft flocked polishing cloth and NX-MET XA05 0.05 μm colloidal silica fused with 20% H_2_O_2_.

To guarantee that the as-received TNZTSF alloy’s composition matched the desired one, an EDS analysis was performed, utilizing a TESCAN VEGA II—XMU XMU scanning electron microscope (SEM) equipped with a BRUKER Quantax xFlash 6/30 EDS detector. The identification of the phases was carried out utilizing X-ray diffraction, employing a RIGAKU MiniFlex600 benchtop diffractometer, which can observe patterns between 30° and 90° in 2θ, and utilizes Cu-Kα radiation, giving limits of detection od roughly 0.1 to 1 wt. % for each phase.

The microstructural analysis was performed using the EBSD technique, utilizing a TESCAN VEGA II—XMU scanning electron microscope (SEM) provided with a BRUKER eFlash1000 EBSD detector. EBSD measurements were carried out while employing the following parameters: 512 × 512 pixel image size, 320 × 240 pixel EBSD resolution, 10 ms acquisition time/pixel, 1 × 1 binning size, and less than 2% zero solutions. Following the EBSD analysis, it could be observed that in all cases, both as-received and cold-rolled, the alloy exhibited a monophasic β-Ti body-centered cubic (BCC) composition, with a lattice parameter of a = 3.304 Å.

Mechanical characterization for both structural states (AR and CR) was carried out using tensile and microhardness testing. The tensile testing was carried out with DEBEN MicroTest-2000N (Deben UK Ltd., Suffolk, UK) testing equipment with a strain rate of 1 × 10^−4^ s^−1^, on “dog bone” tensile test samples with a calibrated area of 2 × 0.8 × 7 mm (with 2 mm, 0.8 mm, and 7 mm being the width, thickness, and gauge length of said area, respectively). The resulting strain–stress curve was utilized in order to obtain some mechanical characteristics, such as yield strength (σ_0.2_), ultimate tensile strength (σ_UTS_), fracture strain (ε_f_) and elasticity modulus (E). The alloy samples were also subjected to microhardness testing, using a SCHIMADZU HMV-2 (Shimadzu, Kawasaki City, Japan) microhardness tester, with the following test parameters: testing force of 100 gf, dwell time of 30 s.

## 3. Results and Discussion

### 3.1. As-Received (AR) State of the TNZTSF Alloy

**Figure 2:**
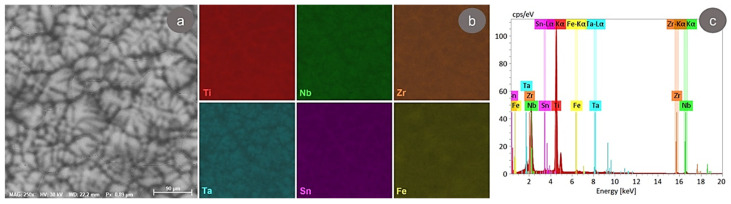
The microstructure of AR TNZTSF alloy (**a**); the distribution mapping of primary alloying elements (**b**); the EDS spectra of the AR TNZTSF alloy (**c**).

The microstructural analysis of the as-received (AR) TNZTSF alloy carried out in this work was conducted using the SEM-EDS technique. A usual SEM-BSE image of the AR TNZTSF alloy is presented in Figure 2a, where it can be seen that the microstructure is made of polyhedral grains, each one presenting an almost uniform distribution of the alloying elements that have high atomic numbers (Z). In Figure 2b is displayed the distribution of the primary alloying elements (titanium, niobium, zirconium, tantalum, tin, and iron) procured with the EDS elemental map, confirming the observation regarding the almost uniform distribution of the alloying elements within the grains. Figure 2c shows the obtained TNZTSF alloy’s EDS spectra, in which one can observe that only the Ti, Nb, Zr, Ta, Sn, and Fe lines are present, with no unwanted alloying elements being present within the TNZTSF alloy’s composition.

The crystalline structure of titanium varies in relation to pressure and temperature, with three solid phases being possible: Ti-α—a hexagonal close-packed structure, this being the low-temperature phase that is stable below 882 °C; Ti-β—a body-centered cubic structure, this being the high temperature phase that is stable between 882 °C and 1670 °C; and Ti-ω—the hexagonal high-pressure phase [16]. The temperature at which titanium makes the transition between the alpha and beta phases is called the beta-transus temperature, and it has a value of 882 °C for pure titanium [17].

This temperature is strongly influenced by the titanium’s purity, with alloying elements being considered as either alpha-stabilizers (Ga, Y, Al, Sn, O) if they increase it, or beta-stabilizers (Mn, Co, Fe, Cu, Ni, Ti, Ta, V) if they decrease it. There are also elements that have little to no effect on the beta-transus temperature, dubbed neutral elements (Sn, Hf, Zr). The beta stability of alloys can be demonstrated by employing the molybdenum equivalency ([Mo]_eq._, wt. %), which uses molybdenum, a beta-stabilizing element, as an arbitrary baseline, and normalizes all other elements to an equivalent molybdenum value (which is positive for beta-stabilizers and negative for alpha-stabilizers) [18,19]:

(1) $${\left[Mo\right]}_{eq.}=1\cdot Mo+0.28\cdot Nb+0.22\cdot Ta+0.67\cdot V+1.6\cdot Cr+2.90\cdot Fe-{\left[Al\right]}_{eq.}$$

(2) $${\left[Al\right]}_{eq.}=1\cdot Al+0.17\cdot Zr+0.33\cdot Sn+10\cdot (O+N)$$

Depending on the value of the molybdenum equivalency, alloys can be considered: α and near-α ([Mo]_eq._ < 2), α + β ([Mo]_eq._ = 2–5), near-β ([Mo]_eq._ = 5–10), β-metastable ([Mo]_eq._ = 10–30) and stable β ([Mo]_eq._ > 30). Considering the TNZTSF alloy’s chemical composition, as presented in Table 1, the calculated value of [Mo]_eq._ is [Mo]_eq._ = 10.22, and this categorizes the TNZTSF alloy as β-metastable ([10 < [Mo]_eq._ < 30).

In Table 1 is displayed the chemical composition of the TNZTSF alloy studied in this work. Since the SEM-EDS technique has some boundaries, the occurrence of a few elements with low atomic numbers (like C or O) was not quantified.

**Figure 3:**
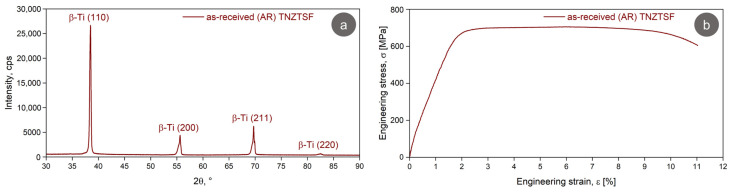
XRD spectra (**a**) and stress–strain curve (**b**) of the TNZTSF alloy in the as-received (AR) state.

Aiming to identify the microstructural constituents of the AR TNZTSF alloy by using the XRD technique, a microstructural characterization was undertaken. Figure 3a displays the acquired XRD spectra of the AR TNZTSF alloy, where it can be seen that the microstructure of our studied alloy consists of only a single phase, namely, Ti-β. The Ti-β phase is indicated by (110), (200), (211), and (220) diffraction lines. The Ti-β phase was indexed in the body-centered cubic (BCC) system, related to the 229/I m-3 m space group. Rietveld analysis showed that the lattice parameter of the Ti-β phase was close to *a*_β_ = 3.304(7) Å, while the internal average microstrain was close to 0.101(3)%.

Looking at the differences between the value of our Ti-β phase lattice parameter (*a*_β_ = 3.304(7) Å) and an ordinary Ti-β phase lattice parameter of around *a*_β_ = 3.282 Å (COD 9,012,924 file), we can come to the conclusion that this increase in lattice parameter may be due to the dissolved alloying elements within the parent Ti-β phase, owing to the elevated atomic radius of the solvable alloying elements (niobium, zirconium, tantalum, tin, and iron).

Figure 3b shows the obtained engineering strain–stress curves of the AR TNZTSF alloy, based on which some mechanical properties like ultimate tensile strength (σ_UTS_), yield strength (σ_0.2_), fracture strain (ε_f_) and elasticity modulus (E) were calculated. From Table 2, where the mechanical properties of the AR TNZTSF alloy are presented, we can infer that the AR TNZTSF alloys show high strength (σ_UTS_ ≅ 705 MPa), moderate ductility (ε_f_ ≅ 11%), and a low elasticity modulus (E ≅ 55 GPa), indicating the suitability of the TNZTSF for use in osseous implantology applications.

**Figure 4:**
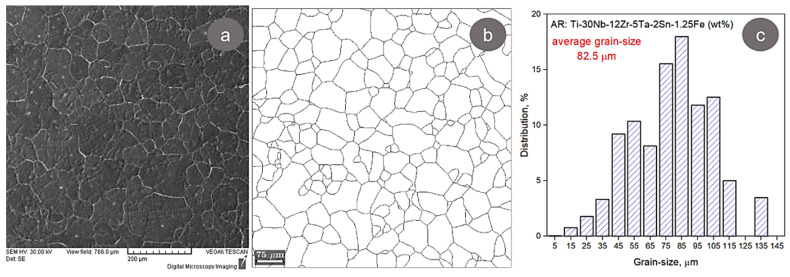
Typical SEM-SE microstructure image (**a**), grain boundary (**b**) and grain size distribution (**c**) of AR TNZTSF alloy.

The microstructural analysis of the AR TNZTSF alloy employing the SEM-SE and SEM-EBSD techniques aimed to morphologically characterize the microstructural constituents. A typical SEM-SE microstructural image is presented in Figure 4a, from which it can be remarked that the AR TNZTSF alloy’s microstructure is composed of polyhedral β-Ti phase grains. The grain boundary analysis (Figure 4b) and the grain-size distribution (Figure 4c) reveal that the AR TNZTSF alloy microstructure is composed of a wide grain-size dispersion of 15 µm to 135 µm. As observed, the largest proportion of grains shows a grain size distribution from 45 µm to 105 µm, with a mean grain size close to 82.5 µm.

### 3.2. Cold-Deformed by Rolling (CR) State of the TNZTSF Alloy

**Figure 5:**
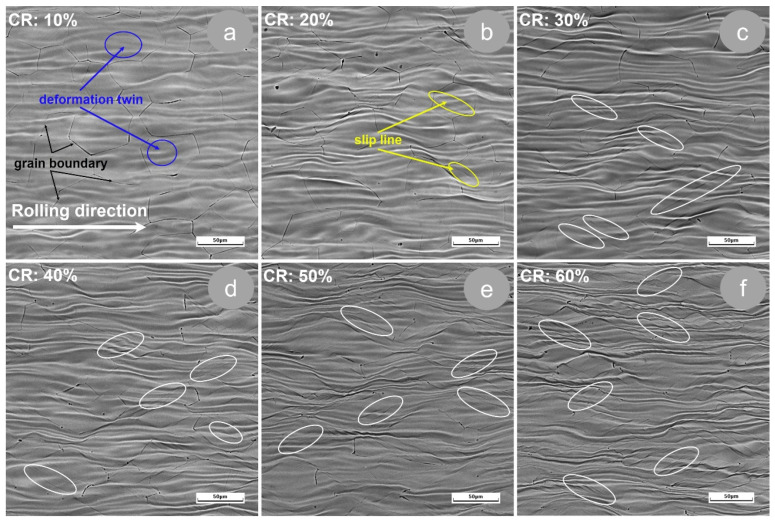
Typical microstructural images of grains’ morphology evolution during cold deformation by rolling (CR) of TNZTSF alloy at ε = 10% (**a**); ε = 20% (**b**); ε = 30% (**c**); ε = 40% (**d**); ε = 50% (**e**); ε = 60% (**f**).

In this work, our TZNTSF alloy was deformed by cold rolling (CR), using deformation degrees ranging from 10% to 60%, with 10% increases. Displayed in Figure 5 are the SEM images of our cold-rolled specimens, as seen from the transversal direction with respect to the rolling direction. As can be seen, after applying intense cold deformation, the initial (AR state) polyhedral equiaxed grains become elongated along the RD processing direction, showing visible deformation bands and deformation twins. Also, it can be remarked that the TNZTSF alloy subject to deformation by cold rolling (CR) did not present discontinuities in volume, even in the case of the most intense applied deformation (60%), proving that the alloy’s microstructure remained homogenous/undamaged. Additionally, it can be noted that, by raising the deformation degree, there is a corresponding increase in the elongation of the initial polyhedral Ti-β grains, with the most obvious deformation-induced microstructural features being seen in Figure 5f, which shows the alloy in its post-60% deformation state. Comparable observations have also been obtained for various types of Ti-alloys [20,21,22,23,24,25,26,27,28,29].

**Figure 6:**
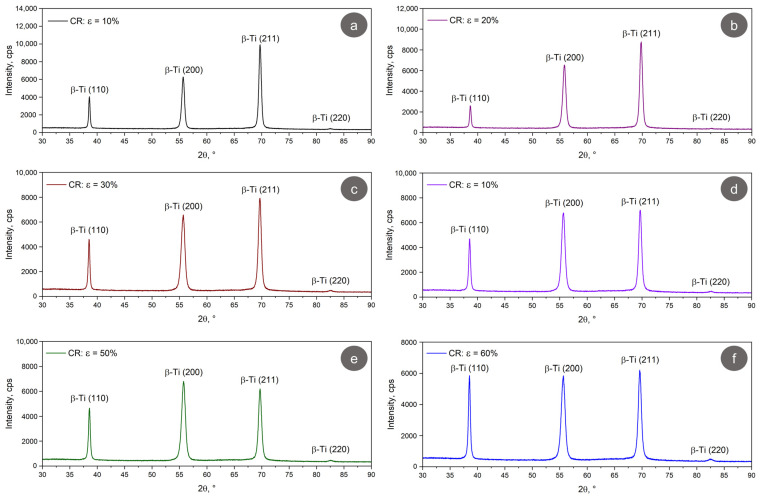
The XRD spectra of the CR TNZTSF at ε = 10% (**a**); ε = 20% (**b**); ε = 30% (**c**); ε = 40% (**d**); ε = 50% (**e**); ε = 60% (**f**).

Figure 6 shows the XRD spectra of our TNZTSF alloy specimens subjected to deformation by cold rolling (CR), utilizing different deformation degrees. Without exception, only the (110), (200), (211), and (220) β-Ti phase diffraction peaks can be seen. As such, no stress-induced martensite phase was formed, meaning that the alloy’s hardness will incur no significant changes as a function of temperature, a useful factor to take into account when considering further thermal processing for the alloy.

Table 3 shows the evolution of the crystallographic parameters of the CR TNZTSF alloy. It is evident that there is only a small variation in the computed lattice parameter, from 3.302(8) Å for a total applied deformation of 10% to 3.312(1) Å for a total applied deformation of 60%, showing that the mechanical processing has no significant impact on the alloy unit cell.

The increase in total applied deformation degree does, however, impact both the crystallite size and the lattice strain, as observed in both Halder–Wagner and Hall models. The lattice strain increases while the crystallite size decreases with an increase in the total applied deformation degree. As observed, the lattice strain increases from 0.19(2)% (Halder–Wagner model)/0.15(4)% (Hall model), for a total applied deformation of 10%, to 0.32(2)% (Halder–Wagner model)/0.47(4)% (Hall model) for a total applied deformation of 60%, while the crystallite size decreases from 542 Å (Halder–Wagner model)/945 Å (Hall model), for a total applied deformation of 10%, to 172 Å (Halder–Wagner model)/187 Å (Hall model), for a total applied deformation of 60%.

Table 4 presents the computed mechanical properties of the TNZTSF alloy specimens after being deformed by cold rolling. It is clear that, with the increase in the total deformation degree, there is a corresponding increase in strength characteristics (ultimate strength, yield strength), with the ultimate strength rising from 1030.7 MPa to 1192.1 MPA, and yield strength rising from 882.6 MPa to 1076.3 MPa (at ε = 10% and ε = 60%, respectively). The rest of the properties (fracture strain, elasticity modulus, microhardness) incur no significant changes with the variation in the total deformation degree. As such, it can be considered that the specimens cold-rolled with a total deformation degree of ε = 60% yield the best mechanical properties.

Analyzing the elasticity modulus, one can observe that a small decrease is observed when one increases the total applied deformation degree, from 57.1 GPa for a total applied deformation of 10% to 54.9 GPa for a total applied deformation of 60%. An opposite behavior is noted in the case of microhardness, which increases from 241 HV0.1 for a total applied deformation of 10% to 249 HV0.1 for a total applied deformation of 60%.

To explain the behavior of mechanical properties with the variation in the total deformation degree, as shown in Table 4, two competing mechanisms can be proffered. The occurrence of UFC/NC grains can be considered as a primary mechanism given that the crystalline grain size is an important factor influencing the mechanical properties, as indicated by the Hall–Petch correlation [28,29]; increased strength properties are the result of a reduction in crystalline grain size. In our work, the smallest crystalline grain size for the β-Ti phase is achieved in the case of the specimens cold-rolled with a total deformation degree of ε = 60%, confirmed by the broad β-Ti peaks achieved for the cold-rolled (CR) state (Figure 6) in contrast to the as-received (AR) state (Figure 3).

The occurrence of a raised dislocation density can be considered as a secondary mechanism, given that these improve the alloy’s strength and at the same time its mechanical properties [30,31,32]. In our work, the highest dislocation density is achieved in the case of the specimens cold-rolled with a total deformation degree of ε = 60%. Both two mechanisms are significantly impacted by the chosen mechanical processing route, the cold-rolled states involving the occurrence of small crystalline grain sizes for the parent β-Ti phase, and higher dislocation density.

Taking into account the demand for implantable biomaterials characterized by a low elasticity modulus (E) [33,34,35,36,37,38,39,40,41,42], the need for an internal microstructure capable of providing this has emerged—a homogenous β-Ti phase microstructure possessing small grain size/crystallite size and high mechanical properties being essential [32,43]. The microstructures/mechanical properties obtained after cold-rolling processing can be further improved by applying different thermal treatments to induce the alloy’s internal microstructure/mechanical properties towards a decreased elasticity modulus (Young’s modulus)/increased strength [44,45,46,47,48,49,50,51,52,53].

Thus, more research should center on identifying the best mechanical processing route that could lead to a further reduction in the elasticity modulus, bringing the values even closer to those found in human bone. Still, the results obtained in this work emphasize that the TNZTSF alloy is a proper candidate for use as a material to be employed in medical implantable applications.

## 4. Conclusions

This work studied the effects of deformation by cold rolling (CR) on the microstructural and mechanical characteristics of a Ti-30Nb-12Zr-5Ta-2Sn-1.25Fe (wt. %) alloy. The primary findings are outlined as follows:

- The Ti-30Nb-12Zr-5Ta-2Sn-1.25Fe (wt. %) alloy, referred to as TNZTSF, was effectively produced through melting in a cold crucible levitation induction furnace;
- The TNZTSF alloy in its as-received (AR) state underwent deformation by cold rolling (CR) using different deformation degrees, ranging from ε = 10% to ε = 60% in 10% increments;
- The microstructure of the TNZTSF alloy, in its as-received (AR) state, consists of a singular, uniform β-Ti phase characterized by equiaxed polyhedral grains with an average size of 82.5 μm, exhibiting a narrow grain size distribution;
- The microstructure of the cold-rolled (CR) TNZTSF alloys also consists of a singular β-Ti phase for all the deformation degrees. The CR TNZTSF alloys displayed a progressive deformation texture, with the most elongated beta Ti grains being caused by the 60% deformation degree. Deformation bands and deformation twins were observed, while no discontinuities in volume were found;
- The crystallographic parameters for both as-received (AR) and cold-rolled (CR) states were quantified based on the XRD spectra analysis. Due to the small variation in the lattice parameter of the CR TNZTSF alloys (3.302(8) Å for a 10% deformation degree to 3.302(1) Å for a 60% deformation degree), it was found that on the alloy unit cells, the mechanical processing had no significant impact. On the other hand, it was shown through Halder–Wagner and Hall models that increasing the deformation degree will impact both the crystallite size and the lattice strain;
- The mechanical properties of the CR TNZTSF alloys compared to the AR TNZTSF alloys have shown major improvements regarding the strength characteristics (ultimate strength (σ_UTS_), yield strength (σ_0.2_));
- The total deformation degree was found to have a visible influence on some mechanical characteristics. By increasing the deformation degree from ε = 10% to ε = 60%, ultimate strength and yield strength will increase, with the ultimate strength rising from 1030.7 MPa to 1192.1 MPa and yield strength from 882.6 MPa to 1076.3 MPa. The variation in the total deformation degree yielded no major influences on the fracture strain (ε_f_) and the microhardness (HV 0.1) values;
- The total deformation degree also has a certain influence on the elasticity modulus (E), a small decrease being recorded with the increase in the deformation degree, from 57.1 GPa for a total applied deformation of 10% to 54.9 GPa for a total applied deformation of 60%;
- The increase in deformation degree from ε = 10% to ε = 60% yields increasingly favorable mechanical properties, with ε = 60% having the best results (high strength/low elastic modulus).

## Figures and Tables

**Table 1:** Quantitative chemical composition of the as-received (AR) TNZTSF alloy.

Element	At. No.	Mass (wt. %)	Mass (at. %)	Abs. Error (%)	Rel. Error (%)
Titanium (Ti)	22	50.27	67.07	1.33	2.71
Niobium (Nb)	41	29.72	20.43	0.77	2.74
Zirconium (Zr)	40	11.87	8.31	0.27	2.85
Tantalum (Ta)	73	4.96	1.75	0.12	3.07
Tin (Sn)	50	1.98	1.07	0.06	3.72
Iron (Fe)	26	1.20	1.37	0.06	3.84
Sum		100.00	100.00		-

**Table 2:** Mechanical properties of the AR TNZTSF alloy.

Structural State	Ultimate Strength, σ_UTS_ (MPa)	Yield Strength, σ_0.2_ (MPa)	Fracture Strain, ε_f_ (%)	Elasticity Modulus, E (GPa)	Microhardness, HV0.1
AR TNZTSF alloy	705.6	658.3	11.1	55.6	226 ± 2

**Table 3:** Crystallographic evolution of the CR TNZTSF alloy.

Structural State	Cold Rolling					
	ε = 10%	ε = 20%	ε = 30%	ε = 40%	ε = 50%	ε = 60%
Lattice parameter, a (Å)	3.302(8)	3.298(3)	3.303(1)	3.306(2)	3.303(9)	3.302(1)
β-Ti(110), 2θ (°)	38.56	38.62	38.49	38.54	38.55	38.50
β-Ti(200), 2θ (°)	55.67	55.78	55.64	55.61	55.71	55.61
β-Ti(211), 2θ (°)	69.68	69.76	69.92	69.63	69.63	69.56
	Halder-Wagner Model					
Lattice strain, ε (%)	0.19(2)	0.21(4)	0.25(4)	0.27(3)	0.31(3)	0.32(2)
Crystallite size, S (Å)	542	447	216	193	181	172
	Hall Model					
Lattice strain, ε (%)	0.15(4)	0.28(1)	0.35(2)	0.41(3)	0.46(2)	0.47(4)
Crystallite size, S (Å)	945	542	442	343	268	187

**Table 4:** Mechanical properties of the CR TNZTSF alloy.

Structural State	Ultimate Strength, σ_UTS_ (MPa)	Yield Strength, σ_0.2_ (MPa)	Fracture Strain, ε_f_ (%)	Elasticity Modulus, E (GPa)	Microhardness, HV0.1
CR: ε = 10%	1030.7	882.6	8.23	57.1	241
CR: ε = 20%	1122.5	1005.5	7.87	56.1	242
CR: ε = 30%	1127.7	1013.7	7.92	56.5	245
CR: ε = 40%	1138.8	1019.5	8.27	55.1	248
CR: ε = 50%	1150.8	1036.6	7.98	56.1	253
CR: ε = 60%	1192.1	1076.3	7.87	54.9	249

## Data Availability

Data are contained within the article.
